# High-intensity interval training improves myocardial work and cardiac function in women with obesity: a randomized controlled study

**DOI:** 10.1038/s41366-026-02158-4

**Published:** 2026-07-10

**Authors:** Caroline Ferraz Simões, Higor Barbosa Reck, João Carlos Locatelli, Gustavo Henrique de Oliveira, Victor Hugo de Souza Mendes, Rogério Toshiro Passos Okawa, Jamie O’Driscoll, Jorge Mota, Wendell Arthur Lopes

**Affiliations:** 1 Research Group on Systemic Arterial Hypertension, Arterial Stiffness and Vascular Aging, Maringa, Brazil; 2 Cesumar University, Maringa, Brazil; 3 Department of Physical Education, State University of Maringa, Maringa, Brazil; 4 School of Human Sciences (Sport Science, Exercise and Health), The University of Western Australia, Perth, WA Australia; 5 Department of Medicine, State University of Maringa, Maringa, Brazil; 6 Diabetes Research Centre, College of Life Sciences, University of Leicester, Leicester, UK; 7 Department of Cardiology, St George’s University Hospitals NHS Foundation Trust,, London, UK; 8 Faculty of Sport, University of Porto, Porto, Portugal; 9 Research Centre for Physical Activity, Health and Leisure, Porto, Portugal

**Keywords:** Randomized controlled trials, Weight management

## Abstract

The purpose of this study was to investigate the effects of high-intensity interval training (HIIT) and moderate-intensity continuous training (MICT) on cardiac function and myocardial work (MW) in women with obesity. Twenty-five women with obesity (Age: 28 ± 5 years, BMI: 35.03 ± 3.19 kg/m²) were randomized to 8 weeks of either HIIT (*n* = 11, 4×4-min at 85–95% of HR_max_) or MICT (*n* = 14, 41-min at 65–75% of HR_max_). Cardiac function and strains were evaluated using two-dimensional speckle tracking echocardiography. MW indices were computed via dedicated software. HIIT significantly improved myocardial efficiency, as evidenced by a reduction in global wasted work (GWW [*d* = 0.64, *p* = 0.042]) and an increase global work efficiency (GWE [*d* = 0.83, *p* = 0.005]). Both HIIT and MICT reduced left ventricular global longitudinal strain (GLS, *p* = 0.017 for HIIT; *p* = 0.023 for MICT), left atrial (LA) contraction (*p* < 0.001 for both), LA conduit (p = 0.002 for both), LA reservoir (*p* = 0.003 for HIIT; *p* < 0.001 for MICT), and LA stiffness (*p* = 0.004 for HIIT, *p* = 0.031 for MICT). HIIT and MICT both improved myocardial deformation in women with obesity. However, only HIIT improved myocardial efficiency, suggesting a potential superiority for targeting subclinical cardiac dysfunction in this population.

**Figure:**
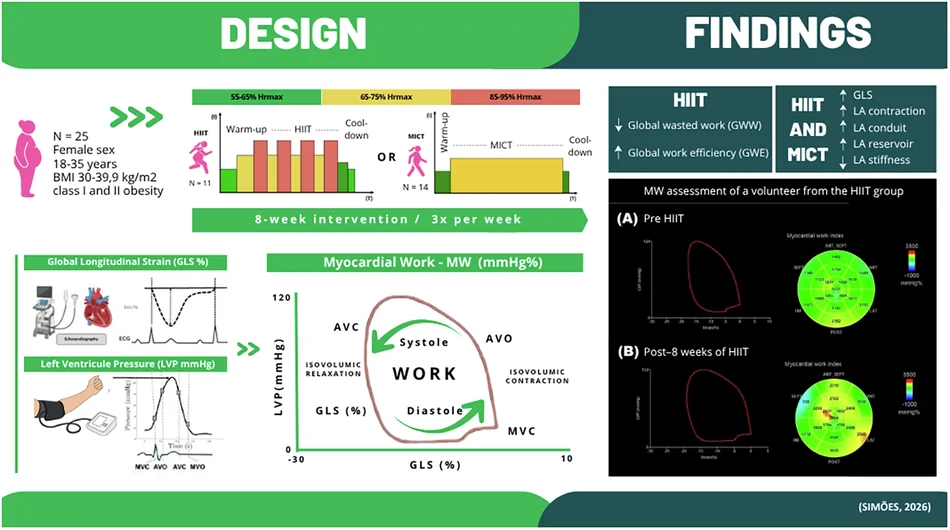

## Introduction

Obesity is characterized by metabolic, inflammatory, and vascular disturbances that increase cardiovascular risk [1, 2]. Even in the absence of overt cardiac disease, individuals with obesity often present subclinical myocardial abnormalities, including impaired strain and altered myocardial work (MW) [3, 4]. Obesity is more prevalent among women, in whom obesity-related cardiovascular consequences appear to be more pronounced, increasing the risk of early myocardial dysfunction [5, 6].

Exercise improves cardiometabolic health independently of significant weight loss [7]. High-intensity interval training (HIIT), characterized by brief bouts of vigorous exercise interspersed with recovery periods [8], is a time-efficient modality that induces robust cardiovascular adaptations and often surpasses moderate-intensity continuous training (MICT) in reducing adiposity and systemic inflammation [9–11]. Meta-analyses also suggest that HIIT elicits greater improvements in vascular function in individuals with obesity compared with MICT [12]. Supporting this evidence, we previously demonstrated greater reductions in central blood pressure following HIIT versus MICT in women with obesity [13] and in other clinical populations [14].

Given that vascular and hemodynamic adaptations may reduce left ventricular afterload, HIIT could also enhance cardiac function and MW in individuals with obesity. Therefore, we investigated the effects of an 8-week HIIT program compared to MICT on cardiac function and MW in women with obesity.

## Materials and methods

Forty-four women with obesity were randomly allocated (1:1) to either HIIT or MICT intervention groups using a computer-generated randomization sequence. Inclusion criteria were: female sex; age 18–35 years; obesity grade I or II (BMI 30.0–39.9 kg/m²); stable body weight for ≥12 weeks; no recent participation in weight-loss programs; absence of cardiovascular, metabolic, or endocrine diseases, except for treated hypothyroidism; non-smoking; no use of medications affecting cardiorespiratory or neuromuscular systems; and availability to complete all assessments.

### Training protocols

Both interventions were performed outdoors on an official running track, three times a week for eight weeks. HIIT consisted of four 4-min bouts at 85–95% HRmax interspersed with 3-min active recovery at 65–75% HRmax. MICT involved 41 min at 65–75% HRmax followed by a 2-min cool-down. Training protocols are detailed in Supplementary Material and Methods.

### Assessments

Echocardiography was performed by a single experienced sonographer blinded to group allocation, with a Vivid E95 system (GE Healthcare Vingmed Ultrasound AS, Horten, Norway). Images were analyzed offline using EchoPAC software (GE Healthcare^®^, Horten, Norway). Systolic blood pressure was measured with an oscillometric device (Omron, Omron Healthcare Brasil, São Paulo, Brazil) immediately before echocardiographic acquisition, and used to compute MW indices. A maximal incremental treadmill test using a ramp protocol assessed VO₂peak and HRmax. Body composition was assessed with a bioelectrical impedance (Maltron® BF-906, Rayleigh, UK). Baseline physical activity levels and dietary intake were assessed using standardized questionnaires. For further details, please see Supplementary Material and Methods.

Participants were instructed to maintain their habitual diet and daily activities and to refrain from initiating any additional exercise programs during the study period. The study was approved by the institutional ethics committee (Protocol: 08935419.5.0000.0104) and registered at the Brazilian Clinical Trials Registry (RBR-3v3dqf). All study procedures were conducted in accordance with the relevant guidelines and regulations. Written informed consent was obtained from all participants prior to their enrollment in the study.

### Data analysis

Sample size was calculated with G*Power® (version 3.1) for a repeated-measures ANOVA, with GLS defined a priori as the primary outcome. Assuming an effect size of 0.51 [15], a power of 80%, and a two-sided *alpha* of 0.05, a minimum of 10 participants was required. Data were analyzed using a per-protocol approach, including only participants who completed the intervention and post-intervention assessments. Data distribution and homogeneity of variances were assessed using the Shapiro–Wilk and Levene’s tests. Continuous data are presented as mean ± standard deviation. Group, time, and group × time effects were analyzed with Bonferroni correction applied when appropriate. Effect sizes were calculated using Cohen’s *d*. Statistical significance was set at *p* ≤ 0.05. Data were analyzed using the SPSS (version 23, IBM®, New York, USA).

## Results

Twenty-five women completed the intervention (HIIT: *n* = 11; MICT: *n* = 14) and were included in the final analysis (Supplementary Table 1). The groups were comparable at baseline for all variables. Mean training attendance was 85%, with no between-group differences.

### Myocardial work indices

**Fig. 1:**
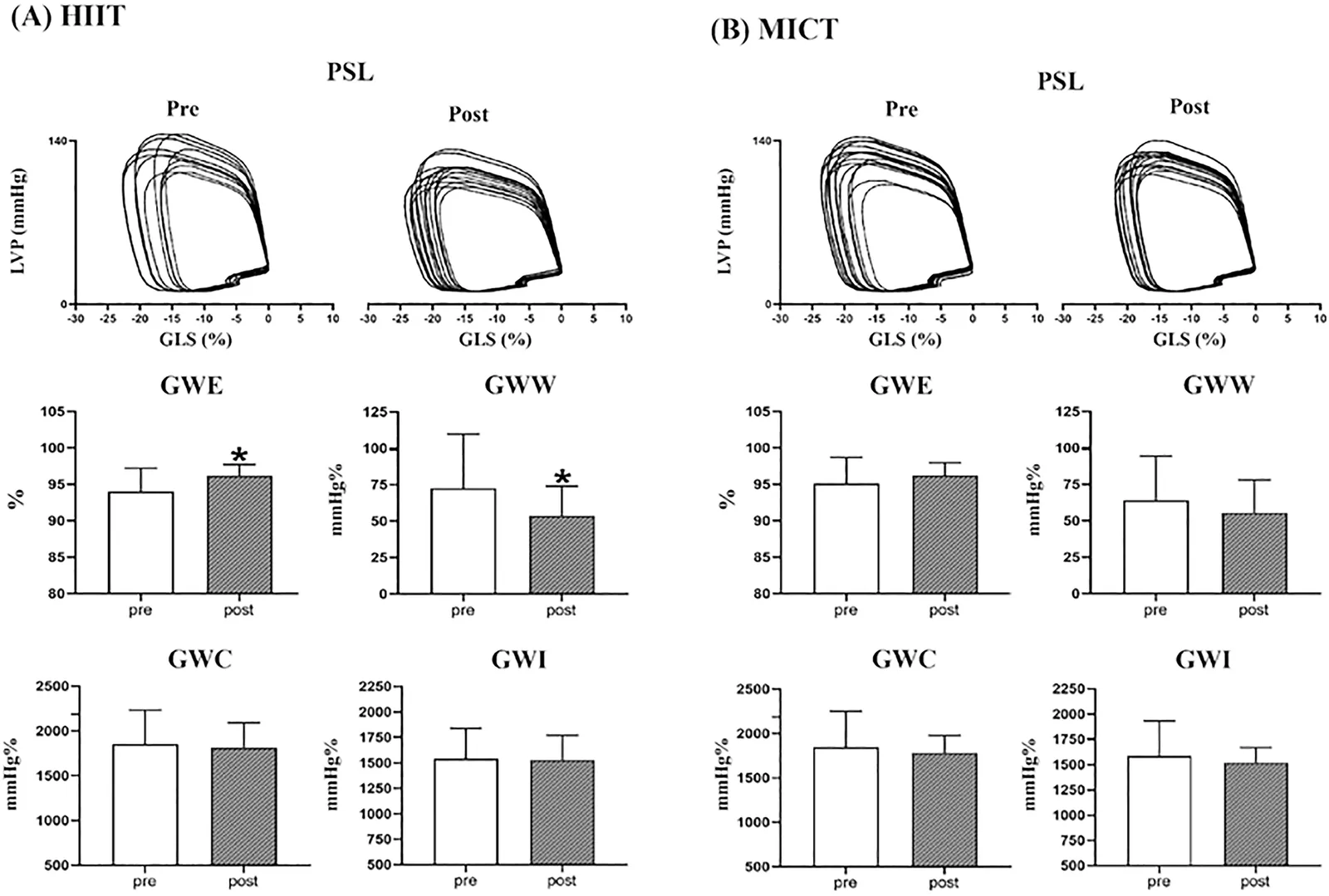
Illustration of pressure–strain loop and myocardial work indices following HIIT (**A**) and MICT (**B**) in women with obesity. PSL pressure-strain loop, LVP left ventricular pressure, GLS global longitudinal strain, GWI global work index, GCW global constructive work, GWW global wasted work, GWE global work efficiency.

No significant group × time interactions were observed in MW indices. A significant within-group effect of time was observed in the HIIT group, with a reduction in global wasted work (GWW Δ = −15.23 ± 41.49 mmHg·%, *p* = 0.042) and an increase in global work efficiency (GWE: Δ= 2.39 ± 2.43%, *p* = 0.005). In contrast, no significant time effects were detected in the MICT group for GWW (Δ= 4.85 ± 62.28 mmHg·%, *p* = 0.283) or GWE (Δ = 1.21 ± 2.75%, *p* = 0.101). No significant time effects were observed in global work index (GWI; HIIT *p* = 0.854; MICT: *p* = 0.292) or global constructive work (GCW; HIIT *p* = 0.646; MICT *p* = 0.396), in any of the groups. Pressure–strain loop (PSL) and MW indices following HIIT and MICT are illustrated in Fig. 1, with a visually larger PSL area observed after HIIT, consistent with improved myocardial efficiency.

### Cardiac function and strain

For conventional cardiac structural and functional parameters, no significant group × time interactions were identified. A significant main effect of time was observed in both groups, with increases in left ventricular end-diastolic volume (LVEDV; HIIT *p* = 0.005; MICT *p* < 0.001) and stroke volume (LVSV *p* < 0.001 for both groups), as well as reductions in left atrial stiffness (HIIT: *p* = 0.004; MICT: *p* = 0.031).

A significant time effect was observed for left ventricular ejection fraction (LVEF; *p* = 0.029) and aortic diameter (*p* = 0.034) only in the HIIT group. No significant time effects were observed in conventional diastolic function parameters in any group (Table 1).

**Table 1:** Effects of 8 weeks of high-intensity interval training (HIIT) and moderate-intensity continuous training (MICT) on body composition, cardiorespiratory fitness, hemodynamics, and cardiac structure and function.

					ANOVA			
Variables	Group	Pre	Post	ES	Time	Group	*Group x Time*	ES
Body composition and cardiorespiratory fitness								
Weight (kg)	HIIT	90.77 ± 14.01	88.8 ± 13.05^a^	0.15	0.001^a^	0.207	0.124	0.61
	MICT	95.43 ± 6.24	94.64 ± 6.13	0.13				
BMI (kg.m^-^²)	HIIT	34.12 ± 3.86	33.35 ± 3.50^a^	0.22	<0.001^a^	0.144	0.129	0.68
	MICT	35.37 ± 2.45	35.32 ± 2.52	0.02				
Body fat (%)	HIIT	44.08 ± 3.62	41.16 ± 3.69^a^	**0.84**	<0.001^a^	0.156	0.054	**0.86**
	MICT	44.86 ± 2.21	43.74 ± 2.19	0.53				
Heart rate (bpm)	HIIT	78.00 ± 9.85	74.09 ± 8.94	0.44	0.055	0.623	0.896	0.29
	MICT	79.43 ± 10.23	76.00 ± 8.69	0.38				
VO_2peak_ (ml kg^−1^ min^−1^)	HIIT	27.50 ± 3.66	29.40 ± 4.93^a^	0.46	0.024^a^	0.150	0.208	0.56
	MICT	25.93 ± 3.63	26.50 ± 3.21	0.18				
Hemodynamic and LV structural variables								
SBP (mmHg^−1^)	HIIT	128.27 ± 12.81	122.00 ± 13.64^a^	0.50	0.021^a^	0.331	0.376	0.41
	MICT	122.57 ± 10.27	119.64 ± 7.66	0.35				
DBP (mmHg^−1^)	HIIT	76.81 ± 11.07	74.00 ± 7.98	0.30	0.007^a^	0.412	0.687	0.17
	MICT	74.85 ± 5.84	71.14 ± 5.50^a^	0.68				
LV Mass (g m^−2^)	HIIT	120.01 ± 19.89	116.09 ± 10.97	0.26	0.949	0.151	0.168	0.60
	MICT	126.64 ± 15.35	130.21 ± 24.12	0.18				
LVED diameter (mm)	HIIT	49.72 ± 3.01	51.36 ± 3.11	0.56	0.028^a^	0.828	0.682	0.18
	MICT	49.71 ± 3.81	50.85 ± 3.05	0.34				
LVES diameter (mm)	HIIT	31.55 ± 2.21	31.36 ± 1.96	0.10	0.822	0.889	0.823	0.09
	MICT	31.78 ± 2.66	31.78 ± 2.63	0.01				
Aorta diameter (mm)	HIIT	28.55 ± 1.97	29.36 ± 1.96^a^	0.51	0.012^a^	0.784	0.517	0.28
	MICT	28.92 ± 2.05	29.42 ± 2.31	0.24				
Systolic function								
LVEF (%)	HIIT	54.18 ± 2.32	56.51 ± 2.85^a^	**0.96**	0.025^a^	0.289	0.281	0.46
	MICT	53.80 ± 2.78	54.71 ± 3.50	0.29				
LVEDV (ml)	HIIT	78.64 ± 10.14	85.0 4 ± 7.95^a^	0.75	<0.001^a^	0.599	0.475	0.31
	MICT	80.14 ± 13.24	88.56 ± 14.94^a^	0.62				
LVESV (ml)	HIIT	36.86 ± 4.89	37.67 ± 4.37	0.18	0.043^a^	0.376	0.228	0.56
	MICT	37.60 ± 5.57	40.64 ± 6.74^a^	0.52				
LVSV (ml)	HIIT	41.78 ± 5.70	47.37 ± 5.03^a^	**1.09**	<0.001^a^	0.829	0.908	0.19
	MICT	42.54 ± 8.52	47.93 ± 10.13^a^	0.60				
S’ septal (m s^−1^)	HIIT	0.09 ± 0.01	0.09 ± 0.01	0.01	0.832	0.758	0.073	0.01
	MICT	0.09 ± 0.01	0.09 ± 0.01	0.01				
S′ lateral (m s^−1^)	HIIT	0.10 ± 0.01	0.11 ± 0.01	**1.00**	0.123	0.707	0.673	0.01
	MICT	0.11 ± 0.03	0.11 ± 0.02	0.01				
Diastolic function								
E (m s^−1^)	HIIT	0.76 ± 0.14	0.79 ± 0.12	0.24	0.157	0.337	0.695	0.03
	MICT	0.72 ± 0.10	0.76 ± 0.13	0.18				
A (m s^−1^)	HIIT	0.53 ± 0.11	0.53 ± 0.09	0.01	0.709	0.057	0.567	0.01
	MICT	0.46 ± 0.06	0.47 ± 0.07	0.16				
E/A ratio	HIIT	1.50 ± 0.45	1.54 ± 0.43	0.10	0.409	0.573	0.857	0.10
	MICT	1.56 ± 0.21	1.62 ± 0.33	0.23				
E′ septal (m s^−1^)	HIIT	0.11 ± 0.02	0.12 ± 0.02	0.01	0.121	0.573	0.308	0.01
	MICT	0.12 ± 0.03	0.12 ± 0.03	0.52				
E′ lateral (m s^−1^)	HIIT	0.15 ± 0.03	0.16 ± 0.03	**0.82**	0.586	0.094	0.147	**0.87**
	MICT	0.18 ± 0.04	0.18 ± 0.04	0.26				
E/e’ ratio	HIIT	6.19 ± 1.17	5.79 ± 1.02	0.38	0.676	0.112	0.083	0.76
	MICT	4.94 ± 0.85	5.57 ± 1.89	0.45				
Cardiac strain								
GLS (%)	HIIT	−*18.87* *±* *2.37*	−20.25 ± 2.36^a^	0.66	0.002^a^	0.468	0.754	0.13
	MICT	−19.63 ± 2.87	−20.78 ± 1.89^a^	0.49				
LASr (%)	HIIT	31.45 ± 9.28	35. 64 ± 5.46^a^	0.64	<0.001^a^	0.599	0.193	0.56
	MICT	28.50 ± 7.44	35.90 ± 5.13^a^	**1.23**				
LASct (%)	HIIT	−13.18 ± 3.16	−16.20 ± 2.42^a^	**1.06**	<0.001^a^	0.454	0.894	0.21
	MICT	−13.86 ± 2.63	−17.00 ± 2.42^a^	**1.45**				
LAScd (%)	HIIT	−21.55 ± 4.16	−23.90 ± 3.81^a^	0.53	<0.001^a^	0.326	0.626	0.08
	MICT	−19.86 ± 3.23	−22.80 ± 3.82^a^	**0.80**				
LA stiffness (%.^−1^)	HIIT	0.21 ± 0.06	0.17 ± 0.03^a^	**0.95**	0.004	0.491	0.388	0.58
	MICT	0.19 ± 0.06	0.16 ± 0.05^a^	0.56				

Data presented as mean ± SD. Bold indicates a large ES.

*ES* effect size, *BMI* body mass index, *VO₂ peak* peak oxygen uptake, *SBP* systolic blood pressure, *DBP* diastolic blood pressure, *LV* left ventricle, *LV mass* left ventricular mass, *LVED* left ventricular end-diastolic, *LVES* left ventricular end-systolic, *LVEF* left ventricular ejection fraction, *LVEDV* left ventricular end-diastolic volume, *LVESV* left ventricular end-systolic volume, *LVSV* left ventricular stroke volume, *S*′ systolic myocardial velocity, *E* early diastolic transmitral velocity, *A* late diastolic transmitral velocity, *E*′ early diastolic myocardial velocity, *GLS* global longitudinal strain, *LASr* left atrial reservoir strain, *LASct* left atrial contractile strain, *LAScd* left atrial conduit strain, *LA* left atrium.

^a^Significant change from baseline within group (*P* < 0.05).

Regarding myocardial deformation, a significant within-group improvement over time was observed in both groups for left ventricular global longitudinal strain (GLS; HIIT *p* = 0.017; MICT *p* = 0.023). Similarly, left atrial reservoir (LASr HIIT: *p* = 0.003; MICT: *p* < 0.001), conduit (LAScd *p* = 0.002 for both), and contraction strain (LASct *p* < 0.001 for both) improved significantly over time in both groups (Table 1).

### Body composition, fitness, and hemodynamics

No significant group × time interactions were detected for body composition or fitness variables. A significant time effect was observed only in the HIIT group, which demonstrated reductions in body weight (*p* = 0.002), BMI (*p* = 0.002), body fat percentage (*p* < 0.001), and systolic blood pressure (SBP *p* = 0.033), along with an increase in VO₂_peak_ (*p* = 0.024). No significant time effects were observed in the MICT group for these outcomes (Table 1).

## Discussion

This study demonstrates that both HIIT and MICT improve cardiac function and LV and LA strain over time in women with obesity. In addition, a significant time effect was observed in MW efficiency, only in the HIIT group, as reflected by a reduction in GWW and an increase in GWE. These findings support the role of aerobic exercise in improving cardiac function and myocardial deformation and suggest that HIIT may confer additional benefits on myocardial efficiency.

Obesity impairs LV mechanics through increased afterload, myocardial fibrosis, and altered metabolic signaling [2–4]. HIIT may counteract these effects by promoting favorable hemodynamic and myocardial adaptations. While prior studies have shown improvements in conventional systolic and diastolic parameters following HIIT [15, 16], our findings extend this knowledge by incorporating MW indices, which integrate myocardial deformation with non-invasively estimated blood pressure, offering a more sensitive and load-adjusted assessment of cardiac performance [17].

Reduced GWW reflects less non-productive myocardial contraction, representing energy expenditure that does not contribute to effective stroke volume, whereas the increase in GWE indicates a greater proportion of effective MW [17]. These changes are clinically relevant, as myocardial inefficiency is an early marker of subclinical dysfunction in obesity [18]. Importantly, a population-based study has shown that increased GWW and reduced GWE independently predict all-cause mortality in individuals with overweight, even when conventional echocardiographic parameters were preserved [19].

The effects of HIIT on MW indices may relate to its intermittent nature, promoting acute increases in cardiac workload, shear stress, and sympathetic activity, stimuli known to enhance myocardial remodeling and vascular function more than MICT [13, 15, 16]. HIIT has been shown to improve endothelial function and reduce arterial stiffness and central blood pressure [12, 14], key factors that lower cardiac afterload and myocardial wall stress, thereby contributing to greater myocardial efficiency [20].

Both interventions improved GLS and LA strain parameters, which are sensitive markers of early myocardial remodeling [21, 22, 23]. These improvements occurred in parallel with increases in left ventricular end-diastolic volume and stroke volume, suggesting that enhanced preload may have contributed to the observed deformation responses. Increased preload enhances myocardial fiber stretch via the Frank–Starling mechanism, thereby improving longitudinal systolic deformation and atrial reservoir, conduit, and contractile function. Therefore, improvements in GLS may reflect not only enhanced subendocardial contractility but also more favorable loading conditions, which are commonly impaired in obesity due to elevated filling pressures and reduced compliance [21]. Similarly, the improvement in LA strain components indicates greater atrial compliance and booster pump function, facilitating left ventricular filling under increased preload and potentially reducing atrial wall stress. These adaptations are clinically relevant, as impaired LA function and reduced preload reserve are early features of obesity-related cardiomyopathy and precede the development of atrial fibrillation and diastolic dysfunction [22].

The significant time effect in reducing body fat and increasing VO₂peak observed only in the HIIT group underscore its superior effectiveness compared to MICT. HIIT promotes greater energy expenditure and metabolic stimulation through mechanisms such as excess post-exercise oxygen consumption and enhanced lipolysis [10]. Additionally, improvements in VO₂peak reflect HIIT’s capacity to induce central and peripheral cardiovascular adaptations [14].

Collectively, these findings suggest that HIIT may represent a time-efficient strategy to improve cardiorespiratory fitness, body composition, and myocardial function and efficiency in women with obesity. The inclusion of MW assessment provides additional load-adjusted insight into myocardial efficiency, allowing a more sensitive evaluation of exercise-induced cardiac adaptations, beyond conventional echocardiographic measures.

Despite the novel findings, this study has important limitations. First, although the sample size was sufficient to detect changes in the primary outcome (GLS), the reduced final sample limits statistical power for secondary outcomes, including MW indices. Second, the high dropout rate (~43%) may have introduced selection bias. However, attrition does not appear to be directly related to the exercise protocols themselves, but may instead reflect sex-specific and contextual barriers to adherence, such as work, family and caregiving demands commonly experienced by women. Third, the inclusion of women only limits generalizability to men, and the absence of a non-exercise control group restricts causal inference. Finally, although physical activity, diet, and medication use were monitored, they were not strictly controlled and may have influenced the outcomes.

Future studies with larger and more diverse cohorts, inclusion of a non-exercise control group, and longer follow-up periods are necessary to confirm these findings and to determine whether improvements in MW translate into clinically meaningful outcomes, such as reduced incidence of heart failure with preserved ejection fraction or atrial fibrillation, in individuals with obesity.

## Supplementary information

Supplemental Material and Methods

Supplementary Figure 1

Supplementary Table 1

## Data Availability

The data supporting the findings of this study can be made available upon reasonable request from the corresponding author.
