# Supplementary material for: High-intensity interval training improves myocardial work and cardiac function in women with obesity: a randomized controlled study
Source: Int J Obes (Lond). 2026 Jul 10;50(10):2248–53. doi: 10.1038/s41366-026-02158-4 (PMC13722585; doi:10.1038/s41366-026-02158-4)
Supplement: Supplementary file 1 [file 41366_2026_2158_MOESM1_ESM.docx]

**Supplementary Material and Methods**

**Clinical assessment**

A comprehensive medical screening based on clinical history, physical examination, and cardiovascular testing, including resting electrocardiography, exercise stress testing, transthoracic echocardiography, and carotid Doppler ultrasonography, was performed to confirm eligibility and exclude participants who did not meet the established criteria.

Dietary intake was assessed at baseline using three 24-hour dietary recalls (two weekdays and one weekend day) [1,2]. Energy and macronutrient intake were analyzed by a registered dietitian using Nutrilife® software (v9.12) and were used for sample characterization.

Physical activity levels were assessed at baseline using the International Physical Activity Questionnaire (IPAQ), which estimated weekly physical activity dose across different activity domains, expressed as METs·min·week⁻¹ [3].

**Training Protocols**

Participants performed either a high-intensity interval training (HIIT) or a moderate-intensity continuous training (MICT) protocol. The HIIT protocol consisted of a 10-min warm-up (5-min at 55–65% of HRmax followed by 5-min at 65–75% of HRmax), four 4-min high-intensity intervals at 85–95% of HRmax interspersed with 3-min active recovery periods at 65–75% of HRmax, and a 5-min cool-down (3-min at 65–75% and 2-min at 55–65% of HRmax), totaling 40-min. The MICT protocol consisted of a 5-min warm-up at 55–65% of HRmax, followed by 41-min of continuous walking or running at 65–75% of HRmax and a 2-min cool-down at 55–65% of HRmax, totaling 48-min.

Heart rate was continuously monitored using a Polar® H10 device (Kempele, Finland) and recorded via Polar Team® software on an iPad® (Apple Inc., USA). Verbal encouragement was provided to ensure adherence to target intensities, and the Borg Rating of Perceived Exertion (RPE) was recorded after each session. Both protocols were matched for energy expenditure [4].

**Echocardiographic Assessment**

All echocardiographic examinations were performed in a quiet, temperature-controlled room after a standardized 10-minute rest period, with participants positioned in the semi-recumbent left lateral decubitus position. Images were acquired at end-expiration using a commercially available ultrasound system (GE Healthcare, Norway) equipped with a 1.4–4.6 MHz transducer. All examinations were conducted by an experienced, accredited sonographer blinded to group allocation and were performed in accordance with the recommendations of the American Society of Echocardiography [5] and the European Association of Cardiovascular Imaging [6]. Three consecutive cardiac cycles were acquired for each view and averaged for analysis. Intra-observer variability was assessed in 10 randomly selected participants. The coefficients of variation for global longitudinal strain (GLS; 3.0%), left ventricular end-diastolic volume (LVEDV; 6.4%), and left ventricular end-systolic volume (LVESV; 7.9%) demonstrated good measurement reliability [7].

*Left Ventricular Structure*

Left ventricular end-diastolic diameter (LVEDd), end-systolic diameter (LVESd), interventricular septal thickness, posterior wall thickness, and aortic root diameter were measured from parasternal long-axis views according to ASE guidelines. Left ventricular mass was calculated using the ASE-recommended formula and indexed to body surface area to obtain LV mass index (g·m⁻²).

*Systolic Function*

Left ventricular end-diastolic volume (LVEDV) and end-systolic volume (LVESV) were measured using the biplane Simpson’s method from apical four- and two-chamber views. Stroke volume was calculated as LVEDV minus LVESV, and left ventricular ejection fraction (LVEF) was derived accordingly. Peak systolic myocardial velocities (S′ septal and S′ lateral) were obtained using pulsed-wave tissue Doppler at the septal and lateral mitral annulus.

*Diastolic Function*

Transmitral early (E) and late (A) diastolic velocities were measured with pulsed-wave Doppler at the mitral leaflet tips, and the E/A ratio was calculated. Early diastolic myocardial velocities (E′) were obtained at the septal and lateral mitral annulus using tissue Doppler imaging. The E/E′ ratio, calculated from the average of septal and lateral E′, was used to estimate left ventricular filling pressures.

*Left Ventricular and Left Atrial Strain*

Global longitudinal strain (GLS) was obtained using two-dimensional speckle-tracking echocardiography from standard apical four-, three-, and two-chamber views, in accordance with ASE/EACVI recommendations. Images were acquired with a frame rate between 60 and 80 frames per second. Myocardial speckles were tracked frame-by-frame using EchoPAC software (GE Healthcare), and GLS was calculated as the mean of 18 left ventricular segments. For inclusion, at least 17 of 18 segments were required to demonstrate adequate tracking quality; segments with persistent tracking failure despite manual adjustment were excluded, and datasets not meeting this criterion were not analyzed. When apical two- and/or three-chamber views were unavailable, GLS was derived from the six segments of the apical four-chamber view. Left atrial endocardial borders were manually traced in apical four- and two-chamber views. Left atrial reservoir (LASr), conduit (LAScd), and contraction (LASct) strain phases were calculated according to EACVI/ASE guidelines. Left atrial stiffness was computed as:  *LA stiffness = (E/E′) / LASr*.

*Myocardial Work Analysis*

Non-invasive myocardial work (MW) indices were calculated using the validated GE Healthcare methodology. Brachial systolic blood pressure (SBP) was measured immediately before image acquisition using a validated oscillometric device (Omron® HEM-7320, Brazil), and the mean of three measurements was used, assuming peak left ventricular systolic pressure equals brachial SBP. Pressure–strain loops were generated by integrating GLS with the estimated left ventricular pressure curve. A standardized pressure curve was adjusted according to valvular timing obtained from Doppler recordings. Mitral valve opening and closure were identified from transmitral Doppler signals, and aortic valve events were obtained from transaortic Doppler. Aortic valve closure was automatically detected by the EchoPAC software based on left ventricular outflow tract Doppler flow and manually adjusted when necessary to ensure correct temporal alignment. The area within the pressure–strain loop was used to calculate the global work index (GWI). Additional indices included global constructive work (GCW), global wasted work (GWW), and global work efficiency (GWE), calculated as GCW/(GCW + GWW) [8].

**Maximal Aerobic Power and Heart Rate**

VO₂peak and HRmax were determined during a maximal incremental treadmill test (Micromed®, Brazil) with open-circuit spirometry (Metalyzer, Cortex®, USA). The protocol began at 3.0 km·h⁻¹ with 1% grade, with increments of 0.35 km·h⁻¹ every 15 s until volitional exhaustion [9]. The test was considered maximal when RPE reached 20 and/or the respiratory exchange ratio was ≥1.10 [10].

**REFERENCES**

1. Majem LS, Bartrina JA, Verdú JM. Nutrición y salud pública: métodos, bases científicas y aplicaciones. 2nd ed. Masson, Barcelona, 1995.
2. Barbosa KBF, Rosado LEFPL, Franceschini SCC, Priore SE. Instrumentos de inquérito dietético utilizados na avaliação do consumo alimentar em adolescentes: comparação entre métodos. Arch Latinoam Nutr. 2007;57:43–50.
3. Matsudo S, Araújo T, Matsudo V, Andrade D, Andrade E, Oliveira LC, et al. Questionário internacional de atividade física (IPAQ): estudo de validade e reprodutibilidade no Brasil. Rev Bras Ativ Fís Saúde. 2001;6:5–18.
4. Rognmo Ø, Hetland E, Helgerud J, Hoff J, Slørdahl SA. High-intensity aerobic interval exercise is superior to moderate-intensity exercise for increasing aerobic capacity in patients with coronary artery disease. Eur J Prev Cardiol. 2004;11:216–22.
5. Mitchell C, Rahko PS, Blauwet LA, Canaday B, Finstuen JA, Foster MC, et al. Guidelines for performing a comprehensive transthoracic echocardiographic examination in adults: recommendations from the American Society of Echocardiography. J Am Soc Echocardiogr. 2019;32:1–64.
6. Lang RM, Badano LP, Mor-Avi V, Afilalo J, Armstrong A, Ernande L, et al. Recommendations for cardiac chamber quantification by echocardiography in adults: an update from the American Society of Echocardiography and the European Association of Cardiovascular Imaging. Eur Heart J Cardiovasc Imaging. 2015;16:233–71.
7. Locatelli JC, Simões CF, Reck HB, Oliveira GH, Mendes VHS, Oxborough D, et al. Acute effects of high-intensity interval training and moderate-intensity continuous training on left ventricular function in women with uncomplicated obesity. Sport Sci Health. 2024;20:1021–31.
8. Samset E. Evaluation of segmental myocardial work in the left ventricle. GE Healthcare, 2017.
9. Jones AM, Doust JH. A 1% treadmill grade most accurately reflects the energetic cost of outdoor running. J Sports Sci. 1996;14:321–27.
10. Binder RK, Wonisch M, Corra U, Cohen-Solal A, Vanhees L, Saner H, et al. Methodological approach to the first and second lactate threshold in incremental cardiopulmonary exercise testing. Eur J Prev Cardiol. 2008;15:726–34.
