# Supplementary material for: High-intensity interval training improves myocardial work and cardiac function in women with obesity: a randomized controlled study
Source: Int J Obes (Lond). 2026 Jul 10;50(10):2248–53. doi: 10.1038/s41366-026-02158-4 (PMC13722585; doi:10.1038/s41366-026-02158-4)
Supplement: Supplementary file 2 [file 41366_2026_2158_MOESM3_ESM.docx]

**Supplementary Table 1**. Baseline participant characteristics.

|  | **TOTAL**  ***N* = 25** | **HIIT**  ***N* = 11** | **MICT**  ***N* = 14** | ***P* value** |
| --- | --- | --- | --- | --- |
| **Demographics** |  |  |  |  |
| Age (yrs) | 28.1 ± 5.0 | 29.4 ± 4.9 | 27.0 ± 5.0 | 0.248 |
| Current smoking, N (%) | 0 (0.0) | 0 (0.0) | 0 (0.0) | 1.000 |
| Asthma, N (%) | 1 (4.0) | 0 (0.0) | 1 (7.1) | 0.365 |
| Hypothyroidism, N (%) | 5 (20.0) | 2 (18.2) | 3 (21.4) | 0.840 |
| **Medication classification** | |  |  |  |
| Thyroid agents, N (%) | 5 (20.0) | 2 (18.2) | 3 (23.1) | 0.840 |
| Bronchodilators agents, N (%) | 1 (4.0) | 0 (0.0) | 1 (7.1) | 0.365 |
| SNRIs/SSRIs, N (%) | 2 (8.0) | 2 (18.0) | 0 (0.0) | 0.096 |
| Contraceptive agents, N (%) | 6 (24.0) | 2 (18.2) | 4 (28.6) | 0.546 |
| **Body composition** |  |  |  |  |
| Weight (kg) | 93.4 ± 10.4 | 90.7 ± 14.0 | 95.4 ± 6.2 | 0.276 |
| BMI (kg.m^-^²) | 35.0 ± 3.2 | 34.1 ± 3.9 | 35.4 ± 2.4 | 0.217 |
| Body fat (%) | 44.1 ± 2.9 | 44.1 ± 3.6 | 44.9 ± 2.2 | 0.193 |
| **Cardiorespiratory fitness** | |  |  |  |
| VO_2peak_ (ml.kg^-1^.min^-1^) | 26.8 ± 3.7 | 27.9 ± 3.7 | 26.3 ± 3.4 | 0.194 |
| Heart rate (bpm) | 186.8 ± 11.5 | 188.1 ± 11.9 | 185.9 ± 11.5 | 0.856 |
| **Hemodynamics** |  |  |  |  |
| SBP (mm.Hg^-1^) | 125.1 ± 11.8 | 128.3 ± 12.8 | 122.6 ± 10.3 | 0.360 |
| DBP (mm.Hg^-1^) | 76.0 ± 8.5 | 76.8 ± 11.1 | 74.8 ± 5.8 | 0.705 |
| Heart rate (bpm) | 78.8 ± 9.9 | 78.0 ± 9.8 | 79.4 ± 10.2 | 0.728 |
| **Physical Activity** |  |  |  |  |
| Walking (MET.min.week⁻¹) | 699.6 ± 631.8 | 711.0 ± 527.4 | 690.6 ± 723.0 | 0.938 |
| MPA (MET.min.week⁻¹) | 403.2 ± 599.6 | 378.2 ± 361.9 | 422.8 ± 749.8 | 0.857 |
| VPA (MET.min.week⁻¹) | 177.6 ± 387.5 | 207.3 ± 394.7 | 154.3 ± 395.0 | 0.742 |
| TPA (MET.min.week⁻¹) | 1280.4 ± 1078.2 | 1296.4 ± 843.1 | 1267.8 ± 1264.6 | 0.949 |
| **Dietary Intake** |  |  |  |  |
| Daily energy intake (kcal) | 1816.8 ± 479.3 | 1726.5 ± 222.0 | 1887.7 ± 625.8 | 0.384 |
| Carbohydrate (%) | 51.3 ± 9.8 | 52.0 ± 10.9 | 50.7 ± 9.3 | 0.759 |
| Lipids (%) | 27.3 ± 9.9 | 25.9 ± 9.9 | 28.3 ± 10.1 | 0.992 |
| Protein (%) | 20.8 ± 6.3 | 20.8 ± 4.1 | 20.8 ± 7.8 | 0.569 |

**BMI**, body mass index; **VO₂peak**, peak oxygen uptake; **SBP**, systolic blood pressure; **DBP**, diastolic blood pressure; **MPA**, moderate physical activity; **VPA**, vigorous physical activity; **TPA**, total physical activity; **MET**, metabolic equivalent; **SNRI**, serotonin–norepinephrine reuptake inhibitor; **SSRI**, selective serotonin reuptake inhibitor.
